# Cigarette Smoking Detection with an Inertial Sensor and a Smart Lighter

**DOI:** 10.3390/s19030570

**Published:** 2019-01-29

**Authors:** Volkan Senyurek, Masudul Imtiaz, Prajakta Belsare, Stephen Tiffany, Edward Sazonov

**Affiliations:** 1 Department of Electrical and Computer Engineering, The University of Alabama, Tuscaloosa, AL 35487, USA; vsenyurek@eng.ua.edu (V.S.); mhimtiaz@crimson.ua.edu (M.I.); pbelsare@crimson.ua.edu (P.B.); 2 Department of Psychology, University at Buffalo, The State University of New York, Buffalo, NY 14260, USA; stiffany@buffalo.edu

**Keywords:** cigarette smoking, hand gestures, IMU sensor, lighter, unobtrusive sensing, wearable sensors

## Abstract

In recent years, a number of wearable approaches have been introduced for objective monitoring of cigarette smoking based on monitoring of hand gestures, breathing or cigarette lighting events. However, non-reactive, objective and accurate measurement of everyday cigarette consumption in the wild remains a challenge. This study utilizes a wearable sensor system (Personal Automatic Cigarette Tracker 2.0, PACT2.0) and proposes a method that integrates information from an instrumented lighter and a 6-axis Inertial Measurement Unit (IMU) on the wrist for accurate detection of smoking events. The PACT2.0 was utilized in a study of 35 moderate to heavy smokers in both controlled (1.5–2 h) and unconstrained free-living conditions (~24 h). The collected dataset contained approximately 871 h of IMU data, 463 lighting events, and 443 cigarettes. The proposed method identified smoking events from the cigarette lighter data and estimated puff counts by detecting hand-to-mouth gestures (HMG) in the IMU data by a Support Vector Machine (SVM) classifier. The leave-one-subject-out (LOSO) cross-validation on the data from the controlled portion of the study achieved high accuracy and F1-score of smoking event detection and estimation of puff counts (97%/98% and 93%/86%, respectively). The results of validation in free-living demonstrate 84.9% agreement with self-reported cigarettes. These results suggest that an IMU and instrumented lighter may potentially be used in studies of smoking behavior under natural conditions.

## 1. Introduction

According to the World Health Organization, smoking is the single most preventable cause of early death [1]. In the world, cigarette smoking causes ten percent of all annual deaths and increases the chances of many serious diseases [2]. The worldwide economic cost of smoking was US $1436 billion in 2012 including direct medical care and lost productivity, equivalent in magnitude to 1.8% of the world’s annual gross domestic product [3]. These statistics underscore the important role of smoking cessation programs [4,5] to promote the economic, social, and, most importantly, health impact of quitting smoking. The first step of these cessation programs is to understand the patient’s smoking pattern over time. The generation of objective data about smoking patterns may enhance the efficacy of smoking cessation programs and contribute useful information about smoking behavior and relapse. The number of cigarettes consumed over a period time or biomarkers such as carbon monoxide and cotinine do not provide sufficient metrics for a detailed examination of smoking behavior. Quantitative measurements of the other metrics of smoking such as number of puffs per cigarette, puff duration, interpuff interval and smoke inhalation volume, besides number of cigarettes, allows researchers to understand the relationship between smoking behavior and smoking cessation [6,7,8]. From self-reporting [9] to portable smoking topography devices [10], many different technologies [11,12,13,14,15,16,17] have been studied to identify powerful and practical ways to monitor smoking activities in daily life.

In recent years, wearable sensor technologies (based on monitoring of lighting events, respiration, hand gestures, etc.) have gained attention in the research of objective monitoring of smoking. A smart cigarette lighter detects when a cigarette is being lit before smoking. However, these lighters only capture and record instances of user’s smoking activity [18,19,20]; no details of the puff characteristics can be found from this information. Respiration sensors measure respiration patterns from chest contractions-expansions and attempt to detect the characteristic breathing patterns associated with smoke inhalation and exhalation [21,22,23,24,25]. However, the respiration sensors are sensitive to motion artifacts, which significantly impact the accuracy of smoking detection. HMGs are prevalent during smoking and may be monitored by several kinds of sensors. The radio frequency-based proximity sensor is a two-part circuit: a receiver on the chest and a transmitter on the wrist (or vice versa) [26]. It detects when the hand is close to the mouth while smoking, however, the strength of the signal depends on the antenna orientation and may not detect all hand gestures. In our previous work that used an earlier version of PACT system, radio frequency-based proximity sensor was used instead of IMU sensor. Smoke inhalations were automatically recognized by using the combination of the proximity sensor and RIP sensor via SVM classifier. The authors reported an F1-score of 83% in lab condition [22]. 

Hand and wrist-mounted Inertial Measurement Units (IMUs) have also been used to identify smoking events from unique hand/arm movements [27,28,29]. IMU-based studies have mainly focused on the HMGs associated with puffs. Although number of HMGs and number of puffs do not match perfectly because of possible multiple puffs within one HMG, research studies [30,31,32] have shown that HMGs can be used as a proxy for the number of puffs. Because of that, in the paper, smoking-HMG detection was mentioned instead of puff detection. 

Most of the previous IMU-based studies use limited datasets: limited number of participants, mostly limited to lab conditions and lacking free-living tests, and limited activity diversity. Some of previous work used more than one IMU device that may be obtrusive to the users.

The goal of the present study was to develop a robust and simple-as-possible sensor-based monitoring solution that can reliably detect smoking events and estimate the puff count for each cigarette irrespective of person, smoking habits, or smoking environment. To accomplish this goal, a new and larger dataset that contains free-living data was created. Then an algorithm was proposed that utilize a wrist IMU device and a smart lighter, two major parts of PACT2.0 [33] sensor platform. We expected the proposed solution to have several advantages over extant research on cigarette smoking detection. First, the wearable sensor system relied on two unobtrusive, simple sensors. Unlike previous studies, a smart lighter was utilized as a supporting tool to reduce false detections. The collaboration of the HMG detection and the lighter event defines a cigarette smoking event. With this approach, the negative effect of mistaken initiation of the lighter was eliminated and a better accuracy was achieved for cigarette smoking detection. Second, we used hand gesture frequency as a means of establishing boundaries of smoking events and used SVM classification to improve the accuracy of HMGs detection and puff count estimation. Third, we performed extensive validation/testing of the sensor system in free-living conditions in the largest study reported to date.

## 2. Related Works

Table 1 summarizes the prior studies on smoking detection using IMU. In [34], the authors described a method by using four 3-axis accelerometers (two at both wrists: dominant hand, dominant upper arm, non-dominant wrist, and ankle). They collected a total of 11.8 h data (34 smoking episodes or 481 puffs) from 6 participants. Using a Random Forest classifier, they reported an F1-score of 70% for puff detection in person-dependent evaluations. However, their performance was 40% for person-independent evaluation. In [35], two 9-axis IMUs (accelerometer, gyroscope, and magnetometer) at the wrist and elbow were used to recognize smoking behavior. The detection model was evaluated with 28 h of data, containing 369 smoking puffs collected from 15 participants. The detection model reached an F1-score of 85% for 10-fold cross-validation. They also applied the model to four users who wore two 9-axis inertial sensors for 4 h each on three days in the field. With this dataset, they reached 83% F1-score. A recent study [36] was conducted on six daily smokers wearing 6-axis IMU on both wrists and a respiration sensor to capture breathing patterns. A total of 291 puffs were collected in these 40 h of data collection. The research achieved a recall of 96.6% and a precision of 87% for smoking. However, this performance was attained by a relatively obtrusive combination of IMU data from the wrist position and Respiratory Inductance Plethysmography (RIP) sensor data from the chest. In [31], 6 participants wore four 6-axis inertial sensors on their dominant arm: one on the wrist, one on the shoulder, and two on the elbow. For each participant, 3.5 h data were collected in a controlled laboratory setting. The authors reported an F1-score between 8% and 86% for different participants. However, use of four IMU devices is obtrusive and not well suited for daily life. In [37], the authors used a smartwatch to detect smoking activity. They collected 45 h of data (17 h for smoking, 28 h for other activities) from 11 participants. The method used in this research for detection of smoking activity achieved an F1-score of 83–94% in a LOSO validation. Unfortunately, the research did not offer information about separate puffs and individual puff duration.

## 3. Materials and Methods

### 3.1. Wearable Sensors

#### 3.1.1. Inertial Sensor

**Figure 1:**
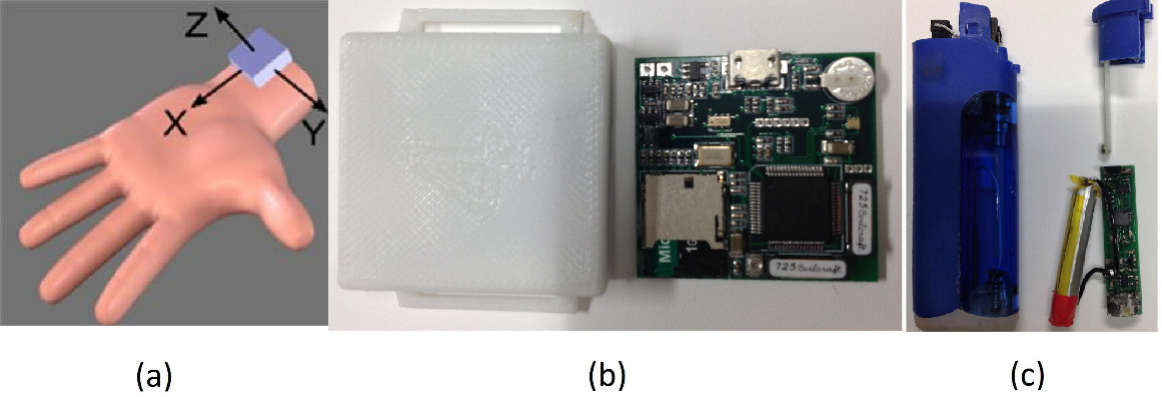
(**a**) Accelerometer axes (positive direction) for hand module, (**b**) Hand module, (**c**) Smart lighter.

The wrist device of PACT v2.0 system [33] was employed for capturing hand gestures associated with both smoking and non-smoking activities. This low-cost module contained a 6-axial IMU (LSM6DS3, STMicroelectronics, Geneva, Switzerland) interfaced with an STM32L151RD processor (STMicroelectronics, Dallas, TX, USA). The sensor data were stored on a 4 GB Micro-SD card accessible via USB interface. The accelerometer and gyroscope were configured to have a ±8 *g* and ±2000 degrees/s measurement range, respectively, with 16 bits of resolution to prevent sensor saturation during sporting activities that can show angular velocity up to 450 degrees/s [38] and acceleration up to several *g*, where $g=9.8\;m/s^{2}$ is the gravitational acceleration. The IMU was sampled at a frequency of 100 Hz. Figure 1 shows the sensor orientation (accelerometer axes) of the hand module placed on the left wrist; this same positioning was also used for right-handed person.

#### 3.1.2. Smart Lighter

The instrumented lighter of a PACT v2.0 system [33] was employed to record the time and duration of all lighting events. This low-cost lighter was a customized version of a commercial piezoelectric lighter. A small magnet was attached to the lighter button to detect trigger events via a Hall Effect sensor mounted inside of the lighter. When the user pressed the trigger button of lighter to light a cigarette, the microcontroller (MSP430G2452, Texas Instruments, Dallas, TX, USA) automatically recorded the event date time on a flash memory. The logged events were accessed through a serial interface.

Both wrist device and smart lighter have an independent clock in. The time across devices needs to be synchronized over the duration of data collection. The initial time synchronization between instrumented lighter and hand modules was established by sending computer time stamp (synchronized with an internet server) using a custom-developed LabVIEW application. At the end of the study, the time of both sensors were read using same application to correct possible drift appeared over the time.

### 3.2. Participants and Study Protocol

A subset of the original study in [35], was used in this study. The original dataset was obtained from medium and heavy smokers who participated in the study between October 2016 and May 2017. The participants signed informed consent. All procedures of the study were approved by the Institutional Review Board at the University of Alabama.

To qualify for the study, participants had to be between the ages of 19–70, report smoking at least 8 cigarettes per day, provide a breath carbon monoxide sample of >10 parts per million (measured using a BreathCO vitalograph [39]), report smoking for >1 years, be healthy and have no acute or chronic respiratory problems. 35 qualifying participants were recruited: 24 men and 11 women; age (average ± standard deviation/range) 25.1 ± 11.8/19–62 year; body mass index 24.6 ± 6.1/16.8–45.9 kg/m^2^; self-reported cigarette consumption 11.4 ± 5.4/5–20 cigarettes per day; and CO measurement 14.7 ± 6.1/ 8–33 ppm).

**Figure 2:**
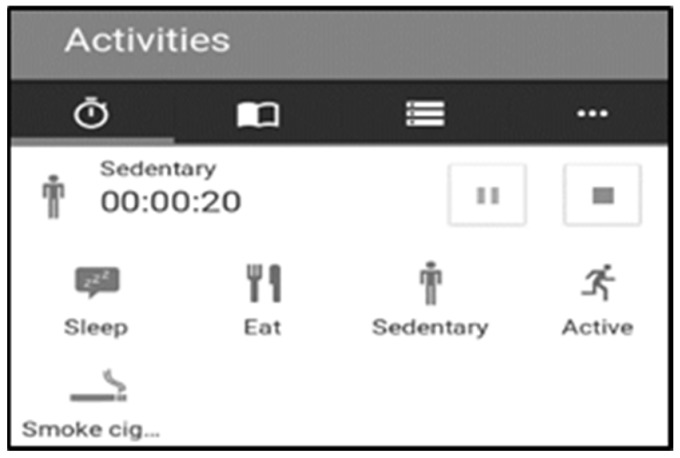
Screenshot of the aTimeLogger application.

The study was composed of a controlled portion (1.5–2 h) at the University of Alabama and an unconstrained free-living portion (~24 h). At the initial visit, participants had been informed with PACT2.0 wearable system, smart lighter and the purpose of the study. Participants had been instructed to smoke as normal with the wearable system. During the controlled portion, the participants were first outfitted with PACT2.0 sensor system. Then participants were asked to perform 10 ordered activities in the lab, outside, and in a cafeteria: 1) read aloud, 2) walk on a treadmill self-selected slow pace (1.8 ± 0.3 mph), 3) walk on a treadmill self-selected fast pace (3.0 ± 0.45 mph), 4) rest by sitting on a chair, 5) smoke while sitting on a chair, 6) talk on cellphone, 7) eat in a cafeteria, 8) smoke while walking and talking, 9) smoke while standing and talking, 10) smoke while walking silently. Activities had a maximum duration of 5 min except for the eating and smoking activities. Between the cigarettes, participants had an unconstrained break of at least 10-min duration. The participants were also free to rest at any time during the experiment. To facilitate annotation of the recorded sensor responses, the entire session was videotaped by an iON camera (Contour Action camera, Provo, UT, USA) time-synchronized with the PACT wrist device sensors. All videotaped data were examined by a research assistant and boundaries of every puff (starting point: the cigarette was put on the mouth, end point: the hand was removed from the mouth) were marked manually to obtain ground truth information. These annotations were used to evaluate detection of smoking-HMGs by computing the number of true positives, false positives and false negatives in the controlled portion of the study. The start and end timestamps of each activity were also marked in a freely available smartphone application (aTimeLogger-Time Tracker). After completion of the controlled study, the participants left the laboratory and started their free-living portion. The activities during free-living portion were not restricted. The participants self-reported major activities (smoking, eating, sleeping, and being sedentary) using a smartphone and aTimeLogger application. All other activities like walking, running, exercise, etc. were self- reported as physically active. Figure 2 shows a screenshot of the aTimeLogger application. Participants returned to the laboratory after 24 h. At the end of visit, participants were asked to participants whether they missed registering any smoking activity in the smartphone. In addition, the participants were asked to complete an ‘acceptability questionnaire’ to evaluate the acceptance of the sensor system in natural conditions. And the subjects scored an average of 8.3 ± 0.31 out of 10 in the acceptability.

### 3.3. Dataset

A total of 871 h of IMU data (55 h of a controlled environment and 816 h of free-living) was recorded by the system from 35 subjects. 463 lighting events (142 in the controlled environment and 321 under free-living conditions) were recorded by the electronic lighter. A total of 303 smoking events were recorded from the users’ self-registration of cigarette consumption during the free-living portion of the study. It was noticed that the duration of some activities was reported as much longer than usual. This shows that some participants may have forgotten to mark the end of some activities. See Table 2 for a brief summary of the dataset.

### 3.4. Signal Preprocessing

The raw IMU data of six dimensions acquired from both controlled and free-living were denoised by a second-order low-pass Butterworth filter with the cutoff frequency of 2 Hz.

### 3.5. Algorithm

**Figure 3:**
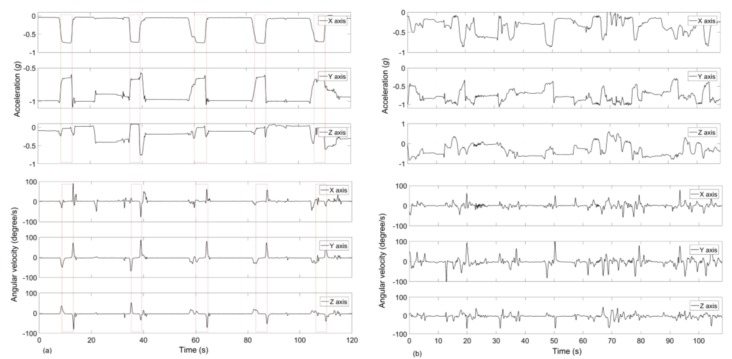
An example of the accelerometer and gyroscope signals from a participant. (**a**) Smoking event, (**b**) an eating event. Dashed lines show the smoking-HMGs.

A characteristic pattern of HMGs, such as an instance of SPM (sequence of puff movement) [31], can be identified sufficiently by using the IMU data stream. Figure 3a illustrates a characteristic pattern in the responses of the accelerometer and gyroscopes axis while a person smoked with the dominant hand. The dashed boxes in the figures indicate the HMGs marked from manual video annotation. When the hand moved toward the mouth, one or two gyroscope axes value increased and reached a peak value; one accelerometer axis value reached a value between ±0.5 *g* and ±1 *g* due to the gravity while the others between 0 *g* and ±0.5 *g*. During a puff, the hand generally remained stationary at the mouth. When the hand moved away from the mouth, gyroscope signals reached the peak value in opposite direction, and the accelerometer values returned to the base level.

Motivated by these characteristic patterns, a complete HMGs detection system is proposed and briefly discussed.

#### 3.5.1. Detection of Hand-to-Mouth Candidate Gestures

**Figure 4:**
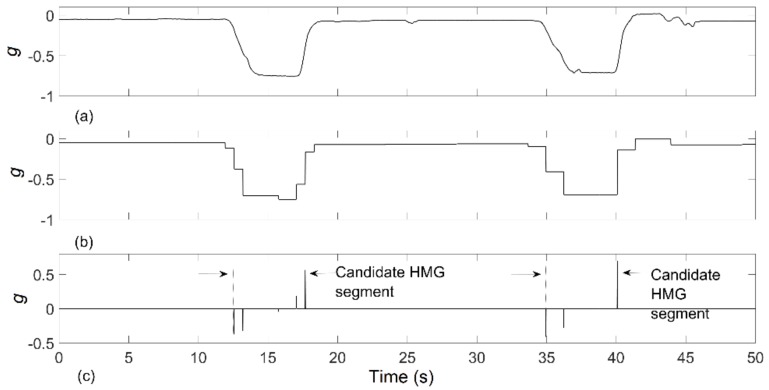
Candidate HMG segments (**a**) *x*-axis accelerometer signal (**b**) wavelet filtered signal (**c**) derivate signal.

An approach was developed to detect smoking related HMG candidates from the raw data collected from *x*-axis accelerometer, which is the parallel axis to the arm. The changes of *x*-axis accelerometer value from high to low indicated the hand was moving towards the mouth. An opposite change in this axis indicated the hand was moving away from the mouth. Every falling and rising edge of the *x*-axis signal was considered as a cigarette HMG candidate. For automatic identification of HMG candidates, an edge-detection algorithm was proposed in the time series (illustrated in Figure 4):

- The accelerometer *x*-axis data was initially filtered by a wavelet filter (Haar type wavelet with nine decomposition levels). Haar wavelet was selected here because of its square shape, which resembles the rapid changes of accelerometer signals.
- A time derivation of the wavelet filtered signal was done to obtain the positive and negative spikes corresponding to signal transitions.
- Spikes with the absolute amplitude smaller than median of positive spikes were discarded to eliminate low-magnitude gestures from further consideration.
- The time segment between of a minimum negative spike and the following maximum positive spike was defined as the HMG candidate.

#### 3.5.2. Feature Extraction and Selection

Initially, 58 features were defined and extracted from the candidate HMG segments. The mean, standard deviations, skewness and kurtosis of accelerometer and gyroscope data, min and max value of the gyroscope signal, HMG duration, time differences between two detected HMGs, pitch and roll angles were considered as features. In the study, roll and pitch angles were calculated as in Equations (1) and (2) without additional filtering to keep the simplicity. These angles can be calculated by using complementary or Kalman filter for better accuracy [40,41]. The accelerometer data of prior and next HMG segments were correlated with current HMG segment, and the correlation coefficients were used as features. In addition, the mean and standard deviation of all accelerometer data from two different durations (3 and 8 s.) of the windows were calculated. The center of HMG segments was defined as the center of these windows.

(1) $$\mathrm{pitch}\left(\theta \right)=\frac{\left(-{\bar{a}}_{x}\right)}{\left({\bar{a}}_{y}\right)}$$

(2) $$\mathrm{roll}\left(\varphi \right)=\frac{\left({\bar{a}}_{z}\right)}{\sqrt{{\bar{a}}_{x}{}^{2}+{\bar{a}}_{y}{}^{2}}}$$

Using too many features may lead to overfitting of the classifier. A large feature set may contain many noise features that do not contribute to the classification. Thus, it is highly important to select a subset of relevant features. Before selection of most relevant features, an optimal number of features as a function of sample size (n) was determined. For this aim, the following process was applied [36]: a) n samples were randomly selected from the controlled portion data, b) a forward selection algorithm was used to find a subset and their misclassification rate based on 10-fold cross-validation. This process was repeated five times, and corresponding error rates were averaged to obtain an estimation of classification error. Gaussian kernel SVM was used; the algorithm stopped when 20 features were determined. 

**Figure 5:**
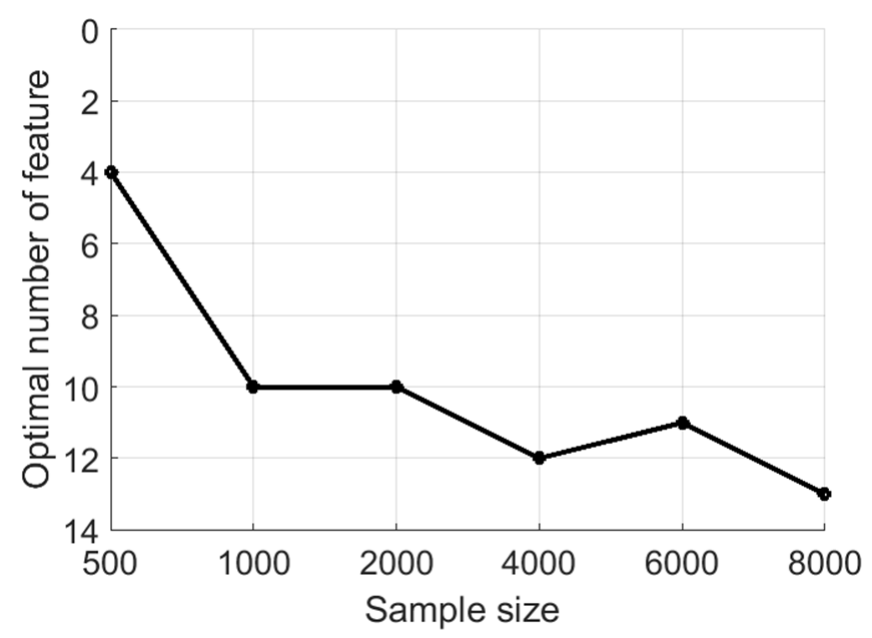
Optimal feature size versus sample size.

The results are shown in Figure 5; the addition of new features did not increase the classification accuracy more than 1% after 12 features, which was determined to be the optimal feature number. The forward selection procedure was used one more time for whole controlled portion data set with 10-fold cross-validation. The algorithm stopped when 12 most relevant features were determined, and these selected features were used for classification (Table 3).

#### 3.5.3. SVM Model

A two-class SVM classifier was employed to detect the smoking HMGs. To provide labels for classification, all 7664 candidate HMGs of the laboratory data were manually labeled as ‘smoking-HMG’ and ‘non-smoking-HMG’ from the video annotations. This procedure identified 1852 as smoking-HMG and 5812 as non-smoking-HMG. The support vector machine was trained from this labeled dataset. To examine whether the proposed method could successfully detect smoking HMGs from a new smoker data set, a LOSO cross-validation method was applied. Data set of 34 participants were used for training of SVM model, and remaining participant’s data were used as the validation set. This procedure was repeated for each participant and 35 models were obtained.

#### 3.5.4. Smoking Event Detection and Reduction of False Positives in Smoking HMGs Detection

**Figure 6:**
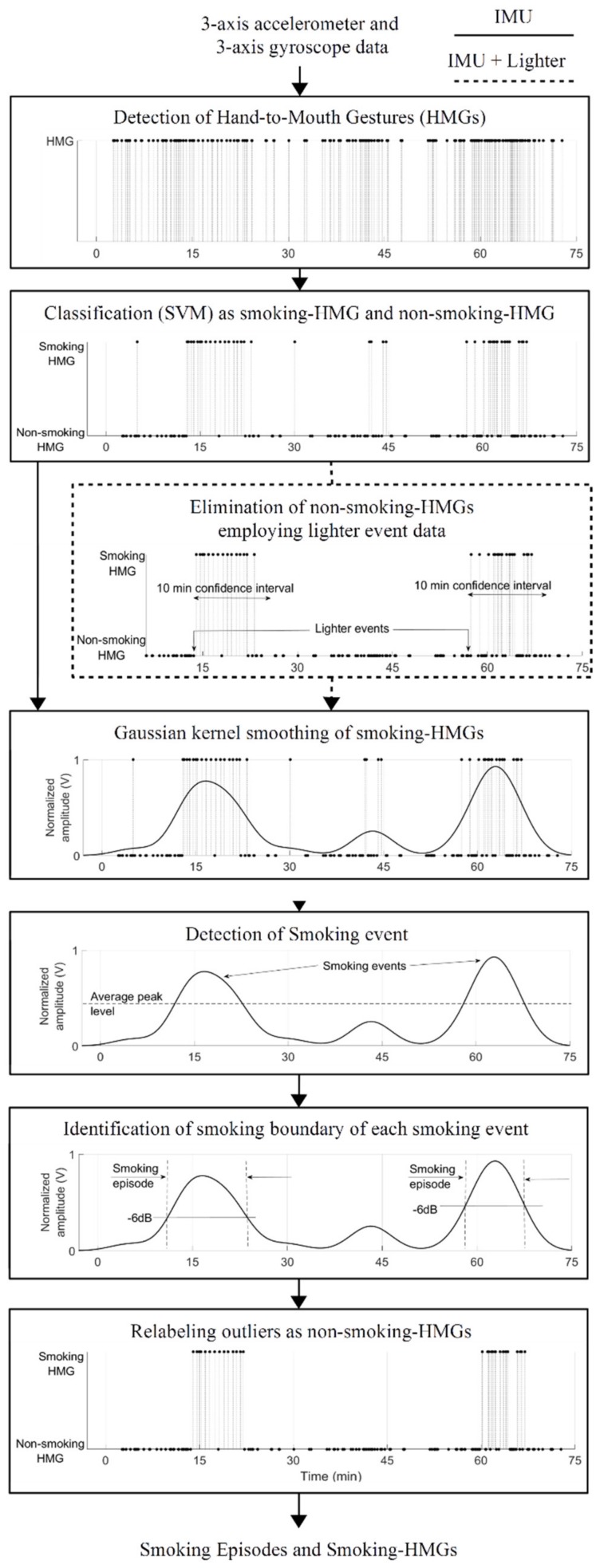
Flowchart of proposed IMU approach for detecting smoking event and smoking-HMG. If a lighter data is available, an extra step indicated by dashed box will be employed for eliminating non-smoking HMGs.

A smoking event was defined as consumption of a full or partial cigarette. The following procedures were applied for smoking event detection. A Gaussian kernel smoothing was used to define a group of smoking-HMGs as a single smoking event. After kernel smoothing, peak points of the kernel smoothing signal were obtained by a peak detection algorithm. Each peak was considered as a center of the smoking event if its level was higher than the average peak level. The start and end of the smoking episodes were determined by using −6 dB bandwidth for each peak. If a detected smoking-HMG was outside of the determined smoking event, it was relabeled as non-smoking-HMG. The instrumented lighter data was used to eliminate non-smoking-HMGs. If a detected HMG was not inside of 10 min confidence interval after the cigarette lighting, it was relabeled as non-smoking-HMG. Then, the boundary of smoking episodes was determined by kernel smoothing. Figure 6 shows the simplified flowchart of proposed approach.

#### 3.5.5. Validation on Free-Living Dataset

In order to obtain person-independent evaluation for the free-living portion, the hold-one-out classifier models obtained from the controlled portion were applied to the free-living data of hold-out subject. That is, model i, which was trained without subject i data, was used on free-living data of subject i. These procedures were repeated for each subject. Self-reports of the participants were used for accuracy computation. A confusion matrix for smoking event detection was calculated along with metrics of average smoking duration per cigarette and average smoking-HMGs number per cigarette. Misclassification rates for each activity of daily living were computed to identify which activity was the main source of error in smoking event detection.

## 4. Results

### 4.1. Results of the Controlled Portion

Table 4 shows HMG and smoking event detection results of for both approaches: using only IMU data, and using IMU with lighter data in the controlled setting. Using only hand IMU data, the average F1-score and accuracy were 77% and 86%, respectively, for HMGs detection. Including the lighter data, these scores reached 86% and 93%, respectively.

**Figure 7:**
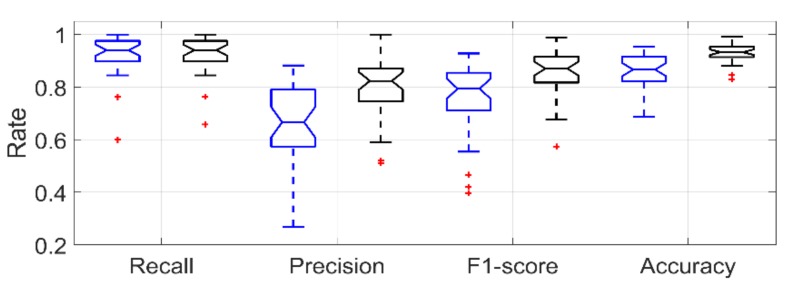
Boxplot of the performance metrics for smoking-HMG detection obtained using LOSO validation in controlled portion. Blue for IMU only, Black for IMU + Lighter.

Figure 7 shows the boxplot for recall, precision, F1-score and accuracy rate for smoking-HMG detection in a LOSO scenario using only IMU data and IMU data with lighter data.

**Figure 8:**
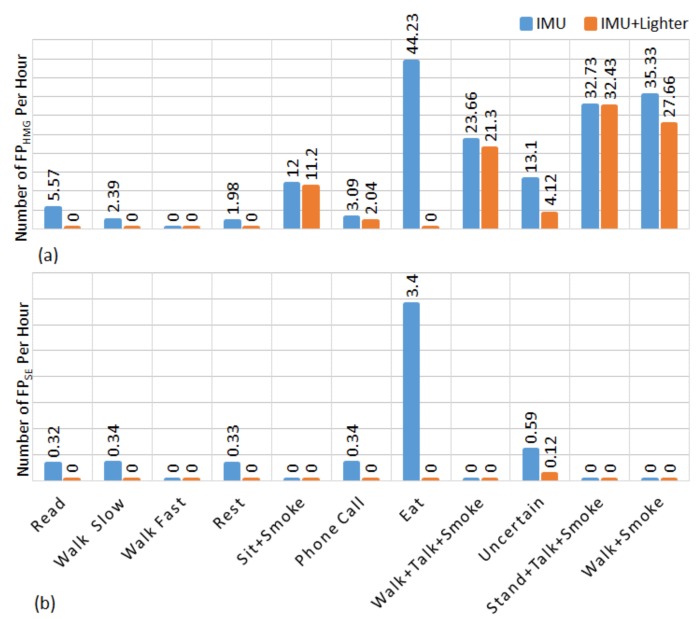
Number of false positive detection per hour in activity for the controlled portion. (**a**) HMGs detection (**b**) smoking events detection. The blue bar belongs to IMU, the yellow one to IMU + Lighter.

In the controlled portion of the study, by using only IMU data a total of 39 false positive smoking events were detected. while 18 of them belongs to eating activity, 17 of them belongs to uncertain activities, other four false positive belongs to reading, slow walking, resting and phone call, respectively. By employing lighter event data, the number of false positive smoking event detection was reduced to three. These three detections belonged uncertain activity. In the controlled portion of the study, the false negative detections belong to the smoking while sitting. Figure 8 shows the number of normalized false positive HMGs (FP_HMG_) and normalized false positive smoking events (FP_SE)_ detection for each activity which were performed in the controlled portion of the study. The start and the end time information of each activity (including walking, talking, eating etc.) was marked in a smartphone application by a researcher. Then this information was used as ground truth information for evaluation and finding statistical analyses. Normalization was done by time spent in activity. In this figure, the uncertain activities are the unconstrained activities during inter-cigarette intervals. This figure indicates that eating is one of the main sources of false detection.

### 4.2. Free-Living Results

**Figure 9:**
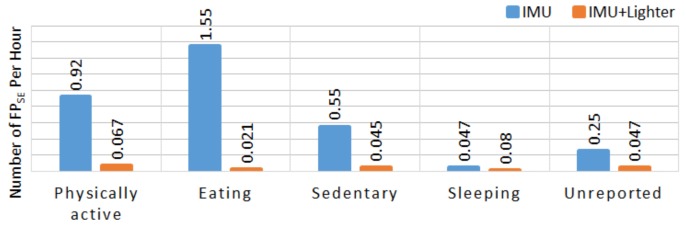
Number of false positive smoking event (FP_SE_) per hour for major activities reported by participants in free-living portion. The blue bar belongs to IMU, the yellow one to IMU + Lighter.

Table 5 shows the number of candidate HMGs and the number of detected smoking-HMG, true negative smoking events (TN_SE_), true positive smoking events (TP_SE_), false negative smoking events (FN_SE_), FP_SE_, and some statistics for the detection of smoking events. The proposed algorithm detected 6354 smoking-HMGs out of 52617 candidate HMGs using only IMU data. By incorporating lighter events (321 lighting events), a total 2641 HMGs were detected as smoking-HMGs. In the free-living portion, the participants reported a total of 303 smoking events. 18 participants reported fewer than seven cigarettes, 13 participants reported between seven and 14 cigarettes, and 4 participants reported more than 20 cigarettes. By comparing the participants’ self-report and the lighter event logs, 26 lighter events occurred outside of the self-report periods. For five subjects, multiple lighter events happened inside one smoking event. No lighter events were detected for 16 self-reported smoking events. By incorporating lighter events, in the free-living portion of the study, a total of 29 false positive smoking events were detected. While nine of them belongs to physical activity, 10 of them belongs to sedentary, one of them belongs to eating, two of them belong to sleeping and seven of them belong to unreported activities. Figure 9 shows the number of FP_SE_ per hour for self-reported activities by the participants using a smartphone application.

## 5. Discussion

The proposed approach validated the efficacy of combining the IMU and electronic lighter for automatic monitoring of smoking. By using only the IMU device in the controlled portion of the study, 77% and 86% F1-score (in the person-independent validation) were achieved for the detection of smoking-HMGs and smoking events, respectively. These metrics were higher than those produced in all previous research. The inclusion of a lighter, however, produced an even higher degree of certainty about the identification of a true smoking event as indexed by the clear improvements of the F1-scores (86% for HMGs, 98% for smoking events).

The dataset of this study recorded unobtrusively, represented realistic smoking behaviors. The participants reported that wearing the wrist device was not obtrusive or concerning. The procedures generated lots of background data to confuse smoking hand movement as non-smoking one. In the controlled portion, the subjects performed four types of smoking activity: sitting + smoking, standing + talking + smoking, walking + talking + smoking and walking + smoking. But smoking during the free-living portion of data collection encompassed unrestricted normal activities of daily living, including those that contain hand gestures similar to smoking.

In the controlled portion of the study, the proposed algorithm detected 230 FP_HMG_ attributable to eating, while 308 FP_HMG_ were associated with uncertain activity. However, subjects spent a total of 5.2 h eating and 23.5 h of unconstrained activity. Figure 8 shows that the most challenging task of the IMU-based approach was to separate smoking HMGs from eating. In terms of smoking events, eighteen of the total FP_SE_ were created by eating activity, whereas fourteen belonged to the uncertain activity. When we visually compare IMU sensor signals belongs to smoking and eating activities, the similarity of hand gesture rate, duration and signal amplitude can be clearly identified (Figure 3).

By employing timestamps from the lighter, non-smoking-HMGs during eating, resting, and cell phone conversation were reduced significantly (Table 4). In the controlled portion, a reduction of FP_HMG_ from 902 to 395 (56.2%), and FP_SE_ from 39 to 3 (92.3%) was achieved. However, some non-smoking-HMGs in a smoking event (which were naturally formed by hand movements during a conversation, scratching head or face etc.) could not be reduced by using lighter data. Also, 3 false smoking events were added by the false press of the lighter by a participant.

For smoking behavior detection in the free-living condition, this research used the largest-to-date dataset with 821 h of sensor data from 35 participants. During the free-living portion, the proposed method detected 6723 smoking-HMGs within 52617 candidate HMGs by using only IMU device. When using the lighter, this number was reduced to 2707 smoking-HMGs. Table 5 shows that the accuracy of free-living smoking detection (F1-score 50%) using only IMU data was low compared to the controlled portion. By including lighter timestamps, smoking events were determined more accurately (91.8% F1-score, 84.9% accuracy); these scores show the lighter provided a powerful tool for reducing FP_HMG_ and FP_SE_.

From the free-living data, a total 29 FP_SE_ were detected. According to the self-report of participants, 22 FP_SE_ belonged to different activities (eating, sedentary, physically active and sleeping) and seven FP_SE_ were detected for the unreported part of the free-living study. There may be several reasons for these false positive detections. The participants might have used the lighter mistakenly when they were physically active, to light items other than cigarettes (e.g., a candle), or to light someone else’s cigarette. Another reason might be that chain-smoking participants did not mark the end of one cigarette and beginning of the next cigarette in self-report. Or might be participants forgot to report their smoking. According to the questionnaire part of the study, 5 participants forgot to report some smoking events.

A total of 21 smoking events was not detected, which could be attributed to errors in self–report or by lighting cigarettes with a different lighter. The proposed approach would be appropriate for regular smokers who are inclined to use the dominant hand for smoking and who are willing to light their cigarettes using the personal lighter. No lighter events were detected for 16 self-reported smoking events. This shows that the subjects may have used their personal lighters or different lighters, instead of the provided instrumented one. Usage of dominant hand was another issue here, which is a clear limitation of the current approach. Employment of another IMU device on the non-dominant hand is a potential option, but that would add an additional burden for the smokers.

By using only the IMU device in the free-living portion, the number of the FP_SE_ was 328 which were associated with eating, sitting, and other activities. Statistics such as average smoking duration and average smoking-HMG number per cigarette provided in the Table 4 and Table 5 provide some possible explanations for this low value of precision. In the control portion, average cigarette consumption duration was computed as 4.7 min and average smoking-HMG number per cigarette was computed as 13. But in the free-living portion, these two parameters changed to 7.5 min and 8.9 smoking-HMG, respectively. These values suggest that the participants consumed cigarettes more quickly with a higher number of HMG in the surveillance condition. But in real life, they consumed more slowly with fewer HMGs. This variation in smoking behavior could affect the accuracy of smoking detection. In the free-living condition, the time between two puffs is longer than during the controlled portion, so the smoking detection algorithm groups these puffs as different smoking events. Also, this smoking behavior negatively affects the recall rate. More sophisticated smoking-HMGs grouping algorithms could potentially improve the detection results for smoking events. Here, the inclusion of the lighter provided a simpler but more accurate solution. Another reason might be that smoking activities that performed in the control portion of the study model did not match with the real-life smoking activities. In the control portion of the study, participants performed four types of smoking activities. But many different types of smoking activities are possible in free-living condition. Although the participants had been instructed to smoke as normal way. Table 4 and Table 5 show the smoking behavior of the participants have changed in the surveillance condition. This problem can solve by training the model using a dataset which has ground truth information and collected in real-life conditions.

Some other issues need to be considered when interpreting the results from the free-living component of the study. For that condition, only an approximate ground truth was available, which was collected by having participants log in their cell phones prior to and after performing daily activities including cigarette smoking. The ground truth was itself subject to errors. For example, 5 participants forgot to report some smoking events. Also, some participants did not report the end time of their activities. Given that self-reports were not consistently veridical with actual smoking behavior, errors of self-report are likely major contributors to the false positive/negative detections in this research. Some FP_SE_ may truly identify non-reported smoking events. Also in the free-living condition, participants were allowed to take off the IMU device when showering or bathing. Some participants may have oriented the system incorrectly when reapplying the device or may have reattached the device too loosely. Either of these issues would have introduced additional error.

The use of the non-dominant hand for smoking or the absence of HMGs during smoking are other possible sources of error. As an example of the latter possibility, users might smoke by holding a cigarette between their lips without any hand movements and thus not generate HMGs. This was not observed in controlled portion of the study, but it could have occurred in free-living conditions.

This study can be compared with two previous studies that that examined smoking behavior under natural conditions. In [30], 4 participants wore two 9D-IMUs, one on the elbow and one on the wrist, for four hours per day for three days. In this limited 48 h dataset, participants reported 30 smoking events. That method detected 27 true events and eight false events and reached 83% F1-score. In [31], breathing pattern was captured from a RIP sensor and hand gestures were captured using a 6D IMU worn on the wrist for puff and lapse (quitting smoking period) detection from newly abstinent smokers. In a free-living condition, the method was applied to 3 days of post-quit data from 32 lapsers. The method detected 28 lapse episode correctly and 14 episodes falsely (75% F1-score). The wrist-only model in that study detected 24 of the 32 lapse events and 49 false episodes (45% F1-score). But the study reported no metrics about detected smoking duration and number of hand gestures.

Accurate analysis of smoking behavior can be possible with quantitative measurement of smoking-related metrics. To achieve this measurement, each smoking-related metrics should be measured by a proper sensor system. For instance, IMU based sensors are appropriate for the number of cigarettes, the number of puff per cigarette and interpuff interval measurement. But, some other metrics such as smoke inhalation volume, smoke holding duration and inhale/exhale duration can be measured by tracking breathing signal via RIP sensor. Another important requirement is the measurement tools or systems should not change the nature of the smoking activity. This is possible with wearable and unobtrusive systems. The main advantage of the system described in the present research, relative to other systems, is that it relies on a simple and low-cost platform that overcomes the limitations of single IMU-based approaches. The evaluation of this system was based on the largest data set reported to date for detection of smoking using IMU technology. The results suggested that the system used in the present research generated better overall detection performance under both controlled and free-living conditions than previously tested systems.

As with any wearable device, the limitation of the proposed system is that the user must wear and use the device (wear the hand gesture sensor and use the instrumented lighter in this case). However, the purpose of the system is to provide objective, accurate information about smoking and smoke exposure. Any wearable system can be defeated by non-compliance. Therefore, we assume compliant users who are interested in cessation.

This study has some other limitations that offer opportunities for further development and refinement of the detection system. First, the system tested in this research used only one wrist device. In future studies, the addition of a second wrist device would allow for the detection of smoking with the non-dominant hand. Second, the PACT wrist device did not have real-time streaming; the data could only be accessed offline. This is one of the weaknesses of the current system for Just-In-Time Adaptive Interventions (JITAIs). The proposed algorithm can be implemented in a smartphone, smartwatch or a cloud-based server to continuously analyze real-time sensor signals if the IMU data are arranged to stream via a Bluetooth or Wi-Fi module. The proposed algorithm could even be implemented to the microcontroller of the wrist device upon applying some optimization algorithm. These tasks are left open for the future. Third, the wearable sensor system used in this research was somewhat bulky and perhaps not well suited for long-term, every-day use. The approach, however, could be extended to smartwatches, as those contain IMUs. Another shortcoming is, a one-day trial in the free-living for each participant is not enough to evaluate test-retest reliability and the effect of the sensor system on smoking behavior.

## 6. Conclusions

This study suggests a practical and reliable method for monitoring cigarette smoking behavior in free-living conditions. The research indicated that the combination of an IMU sensor with an instrumented lighter provides better results for smoking behavior analysis than reported in previous studies examining smoking detection. In the controlled portion of the research, the system achieved a very high accuracy in the person-independent scenario for smoking-HMG (86% F1-score) detection and smoking event (98% F1-score) detection. Under free-living conditions, the proposed method achieved 91% F1-score and 84% accuracy. These findings provide a foundation for a wide variety of applications and suggest that this approach can be used in a range of studies to provide accurate, bias-free measurements of smoking behavior in free-living conditions.

## Figures and Tables

**Table 1:** Related work on smoking monitoring employing inertial sensors.

Study	[34]	[36]	[31]	[35]	[37]	This Study
**IMU Type**	3D	6D	6D	9D	6D	6D
**IMU No**	4	1	4	2	1	1
**Extra Sensor**		RIP				Lighter
**Classifier**	Random Forest	SVM	SVM, Edge Detector	Conditional Random Forest	Hierarchical	SVM
**Validation Procedure**	5-fold	10-fold		10-fold & LOSO	LOSO	LOSO
**Performance** **(F1-score)**	0.70 for HMG, 0.79 for smoking	0.91 for HMG	0.08–0.86 for HMG	0.85 for HMG	0.83–0.94 for smoking	0.86 for HMG, 0.98 for smoking
**Activities^1^**	S + E, S + W, S + T T, D, St	S + St, S + T, S + St, S + Si	S + Si, W, C S + R	S + St, S + T, S + W, E, D	S + St, S + Si, S + T, E, Si, W, St	R, W, Si, Si + S, St + T+ S, C, E, W + T + S, W + S
**Subjects**	6	6	6	15 lab, 4 Wild	11	35
**Length of dataset (hr)**	11.8	40	21	28, 48 for wild	45	55,816 for wild
**Study Type**	Lab.	Lab. & Wild	Lab.	Lab. &Wild	Lab.	Lab. &Wild
**Detection**	HMG, Smoking	HMG, Lapse	HMG, Smoking	HMG, Smoking	Smoking	HMG, Smoking

^1^ Activities: S = Smoking, St = Standing, Si = Sitting, T = Talk, E = Eating, D = Drink, W = Walk, C = Using Cellphone, R = Reading.

**Table 2:** Data set of the study.

Dataset			
	**Lab.**	**Free-living** **^1^**	
Number of Participants	35	35	
Test duration	1.5–2 h	~24 h	
Total duration	55 h	816 h	
Number of lighting events	142	321	
Number of smoking events	140	303	
Number of puffs	1852	-	
Ground truth	Smoking events and puffs	Smoking events	
Total activity durations (h)			
Eating	5.2	Eating	23.2
Reading	3.05	Sedentary	218
Slow walking	2.93	Sleeping	252
Fast walking	2.83	Smoking	44.8
Phone calling	2.91	Physically Active	130
Sitting + Smoking	2.5	Unreported	148
Walking + Talking + Smoking	3.38		
Standing + Talking + Smoking	3.33		
Walking + Smoking	2.3		
Resting	3.03		
Uncertain activity	23.5		

^1^ According to the self-report of participants.

**Table 3:** Selected features.

Selected Features		
**Time features**	**Number**	
Durations	2	Detected HMG duration, the time difference between current and prior detected HMGs.
**Accelerometer Features ^1^**		
Accelerometer *x*-axis	3	Correlation coefficients between prior and current, next and current HMG period, mean of 8 s sizes of window data
Accelerometer *y*-axis	2	Kurtosis, Correlation coefficient between next and current HMG period
Accelerometer *z*-axis	2	Mean, the standard deviation of 3 s sizes of window data
**Gyroscope Features ^1^**		
Gyroscope *x*-axis	1	Standard deviation
Gyroscope y-axis	1	Maximum
Gyroscope *z*-axis	1	Standard deviation

^1^ Features were computed over each hand gesture duration.

**Table 4:** HMG and smoking event detection results for controlled portion.

		TN	FP	FN	TP
HMG detection	IMU	4910	902	128	1724
	IMU + Lighter	5417	395	126	1726
Smoking event detection	IMU	0	39	3	137
	IMU + Lighter	0	3	1	139
		**Ground truth**		**Detected (IMU + Lighter)**	
Number of cigarettes		140		142	
Number of smoking-HMGs per cigarette		13.01 (±5.5)		15.04 (±6.1)	
Smoking duration (min)/cig. Average, (SD)		4.7 (±1.4)		5.6 (±1.96)	

**Table 5:** Free-living test results.

HMG Detection												
				Candidate HMGs						Detected smoking-HMGs		
Only IMU data				52,617						6723		
IMU data + lighter				52,617						2707		
**Smoking event detection**												
	TN_SE_		FP_SE_		FN_SE_	TP_SE_	Recall	Precision			F1-score	Accuracy
Only IMU data		0	328		103	216	0.677	0.397			0.5	0.333
IMU data + lighter		0	29		21	282	0.93	0.906			0.918	0.849
						**Ground truth^1^**			**Detected (IMU + Lighter)**			
Number of cigarettes						303			311			
Number of smoking-HMG per cigarette						-			8.9 (±5.2)			
Smoking duration(min)/cig. Average, (SD)						8.3 (±7.8)			7.5 (±1.5)			

^1^ According to the self-report of participants.

## References

[1] World Health Organization (2012). WHO global report on mortality attributable to tobacco. WHO Global Report on Mortality Attributable to Tobacco.

[2] World Health Organization (2017). WHO Report on the Global Tobacco Epidemic, 2017: Monitoring Tobacco Use and Prevention Policies.

[3] Goodchild M., Nargis N., d’Espaignet E.T. (2018). Global economic cost of smoking-attributable diseases. Tobacco Control.

[4] Ghorai K., Akter S., Khatun F., Ray P. (2014). mHealth for smoking cessation programs: A systematic review. J. Personal. Med..

[5] Mañanes G., Vallejo M.A. (2014). Usage and effectiveness of a fully automated, open-access, Spanish Web-based smoking cessation program: Randomized controlled trial. J. Med. Internet Res..

[6] Blank M.D., Disharoon S., Eissenberg T. (2009). Comparison of methods for measurement of smoking behavior: Mouthpiece-based computerized devices versus direct observation. Nicotine Tobacco Res..

[7] Vinci C., Haslam A., Lam C.Y., Kumar S., Wetter D.W. (2018). The use of ambulatory assessment in smoking cessation. Addict. Behav..

[8] Kim S., Yu S. (2018). Smoking Topography among Korean Smokers: Intensive Smoking Behavior with Larger Puff Volume and Shorter Interpuff Interval. Int. J. Environ. Res. Public Health.

[9] Velicer W.F., Prochaska J.O., Rossi J.S., Snow M.G. (1992). Assessing outcome in smoking cessation studies. Psychol. Bull..

[10] Lee E.M., Malson J.L., Waters A.J., Moolchan E.T., Pickworth W.B. (2003). Smoking topography: Reliability and validity in dependent smokers. Nicotine Tobacco Res..

[11] Shiffman S. (2014). Conceptualizing Analyses of Ecological Momentary Assessment Data. Nicotine Tobacco Res..

[12] Kalkhoran S., Glantz S.A. (2016). E-cigarettes and smoking cessation in real-world and clinical settings: A systematic review and meta-analysis. Lancet Respiratory Med..

[13] Stennett A., Krebs N.M., Liao J., Richie J.P., Muscat J.E. (2018). Ecological momentary assessment of smoking behaviors in native and converted intermittent smokers. Am. J. Addict..

[14] Spohr S.A., Nandy R., Gandhiraj D., Vemulapalli A., Anne S., Walters S.T. (2015). Efficacy of SMS text message interventions for smoking cessation: A meta-analysis. J. Substance Abuse Treat..

[15] Odetallah A.D., Agaian S.S. Human visual system-based smoking event detection. Proceedings of the SPIE Defense.

[16] Shiffman S., Kirchner T.R., Ferguson S.G., Scharf D.M. (2009). Patterns of intermittent smoking: An analysis using Ecological Momentary Assessment. Addict. Behav..

[17] Rakshit R., Khasnobish A., Chowdhury A., Sinharay A., Pal A., Chakravarty T. (2018). A Novel Approach to the Identification of Compromised Pulmonary Systems in Smokers by Exploiting Tidal Breathing Patterns. Sensors.

[18] Scholl P.M., Kücükyildiz N., Laerhoven K.V. When do you light a fire? Capturing tobacco use with situated, wearable sensors. Proceedings of the 2013 ACM Conference on Pervasive and Ubiquitous Computing Adjunct Publication.

[19] Scholl P.M., Van Laerhoven K. Lessons learned from designing an instrumented lighter for assessing smoking status. Proceedings of the 2017 ACM International Joint Conference on Pervasive and Ubiquitous Computing and Proceedings of the 2017 ACM International Symposium on Wearable Computers.

[20] Quitbit Smart Lighters to Help Track, Reduce & Quit Smoking. http://www.quitbitlighter.com/index.

[21] Lopez-Meyer P., Tiffany S., Sazonov E. Identification of cigarette smoke inhalations from wearable sensor data using a support vector machine classifier. Proceedings of the 2012 Annual International Conference of the IEEE Engineering in Medicine and Biology Society (EMBC).

[22] Lopez-Meyer P., Tiffany S., Patil Y., Sazonov E. (2013). Monitoring of cigarette smoking using wearable sensors and support vector machines. IEEE Trans. Biomed. Eng..

[23] Ali A.A., Hossain S.M., Hovsepian K., Rahman M.M., Plarre K., Kumar S. mPuff: Automated detection of cigarette smoking puffs from respiration measurements. Proceedings of the 2012 ACM/IEEE 11th International Conference on Information Processing in Sensor Networks (IPSN).

[24] Ramos-Garcia R.I., Sazonov E., Tiffany S. Recognizing cigarette smoke inhalations using hidden Markov models. Proceedings of the 2017 39th Annual International Conference of the IEEE Engineering in Medicine and Biology Society (EMBC).

[25] Sazonov E., Lopez-Meyer P., Tiffany S. (2013). A wearable sensor system for monitoring cigarette smoking. J. Stud. Alcohol Drugs.

[26] Lopez-Meyer P., Patil Y., Tiffany T., Sazonov E. (2013). Detection of hand-to-mouth gestures using a RF operated proximity sensor for monitoring cigarette smoking. Open Biomed. Eng. J..

[27] Scholl P.M., Van Laerhoven K. A feasibility study of wrist-worn accelerometer based detection of smoking habits. Proceedings of the 2012 Sixth International Conference on Innovative Mobile and Internet Services in Ubiquitous Computing (IMIS).

[28] Varkey J.P., Pompili D., Walls T.A. (2012). Human motion recognition using a wireless sensor-based wearable system. Pers. Ubiquit. Comput..

[29] Shoaib M., Bosch S., Incel O., Scholten H., Havinga P. (2016). Complex Human Activity Recognition Using Smartphone and Wrist-Worn Motion Sensors. Sensors.

[30] Parate A., Ganesan D., Rehg J.M., Murphy S.A., Kumar S. (2017). Detecting Eating and Smoking Behaviors Using Smartwatches. Mobile Health: Sensors, Analytic Methods, and Applications.

[31] Raiff B.R., Karataş Ç., McClure E.A., Pompili D., Walls T.A. (2014). Laboratory validation of inertial body sensors to detect cigarette smoking arm movements. Electronics.

[32] Sazonov E., Metcalfe K., Lopez-Meyer P., Tiffany S. RF hand gesture sensor for monitoring of cigarette smoking. Proceedings of the 2011 Fifth International Conference on Sensing Technology (ICST).

[33] Imtiaz M., Ramos-Garcia R., Senyurek V., Tiffany S., Sazonov E. (2017). Development of a Multisensory Wearable System for Monitoring Cigarette Smoking Behavior in Free-Living Conditions. Electronics.

[34] Tang Q., Vidrine D.J., Crowder E., Intille S.S. Automated detection of puffing and smoking with wrist accelerometers. Proceedings of the 8th International Conference on Pervasive Computing Technologies for Healthcare.

[35] Parate A., Chiu M.-C., Chadowitz C., Ganesan D., Kalogerakis E. Risq: Recognizing smoking gestures with inertial sensors on a wristband. Proceedings of the 12th Annual International Conference on Mobile Systems.

[36] Saleheen N., Ali A.A., Hossain S.M., Sarker H., Chatterjee S., Marlin B., Ertin E., Al’Absi M., Kumar S. puffMarker: A multi-sensor approach for pinpointing the timing of first lapse in smoking cessation. Proceedings of the 2015 ACM International Joint Conference on Pervasive and Ubiquitous Computing.

[37] Shoaib M., Scholten H., Havinga P.J., Incel O.D. A hierarchical lazy smoking detection algorithm using smartwatch sensors. Proceedings of the 2016 IEEE 18th International Conference on e-Health Networking, Applications and Services (Healthcom).

[38] Yeadon M.R., King M.A., Wilson C. (2006). Modelling the maximum voluntary joint torque/angular velocity relationship in human movement. J. Biomech..

[39] Vitalograph—BreathCO. https://vitalograph.com/product/162449/breathco.

[40] Sabatini A.M. (2006). Quaternion-based extended Kalman filter for determining orientation by inertial and magnetic sensing. IEEE Trans. Biomed. Eng..

[41] Szczęsna A., Skurowski P., Lach E., Pruszowski P., Pęszor D., Paszkuta M., Słupik J., Lebek K., Janiak M., Polański A. (2017). Inertial Motion Capture Costume Design Study. Sensors.

